# Wall Properties of Elastic and Muscular Arteries in Children and Adolescents at Increased Cardiovascular Risk

**DOI:** 10.3390/jcm12216919

**Published:** 2023-11-03

**Authors:** Simonetta Genovesi, Elena Tassistro, Giulia Lieti, Ilenia Patti, Marco Giussani, Laura Antolini, Antonina Orlando, Paolo Salvi, Gianfranco Parati

**Affiliations:** 1 Department of Cardiology, Istituto Auxologico Italiano, IRCCS, 20100 Milan, Italy; 2 School of Medicine and Surgery, University of Milano-Bicocca, 20100 Milan, Italy

**Keywords:** adolescent, arterial stiffness, blood pressure, body mass index, carotid-femoral pulse wave velocity, carotid-radial pulse wave velocity, children, HOMA index, waist-to-height ratio

## Abstract

Background: Pulse wave velocity (PWV) assessment represents a simple method to estimate arterial distensibility. At present, carotid-femoral PWV (cf-PWV) is considered the gold standard method in the non-invasive evaluation of the elastic properties of the aorta. On the other hand, the mechanical properties of muscular arteries can be evaluated on the axillo-brachial-radia axis by estimating the carotid-radial PWV (cr-PWV). While a number of studies have addressed these issues in adults, limited information is available on the respective features of cf-PWV and cr-PWV and on their modulating factors in children and adolescents at increased cardiovascular risk. Methods: The mechanical properties of the predominantly elastic (aorta) and muscular (axillo−brachial−radial axis) arteries were evaluated in a pediatric population characterized by either elevated blood pressure (BP) or excess body weight, and the main factors affecting cf-PWV and cr-PWV values in these individuals were investigated. Results: 443 children and adolescents (median age 11.5 years, 43.3% females) were enrolled; 25% had BP values >90th percentile and 81% were excess weight. The cf-PWV values were significantly lower than the cr-PWV values: median (Q1–Q3) = 4.8 m/s (4.3–5.5) and 5.8 m/s (5.0–6.5), respectively (*p* < 0.001). The pubertal development (*p* < 0.03), systolic BP and diastolic BP z-scores (*p* = 0.002), heart rate (*p* < 0.001), and waist-to-height ratio (*p* < 0.005) were significantly associated with cf-PWV values. No significant association was found between BMI z-score and cf-PWV. Predictors of high cf-PWV (>95th percentile) were the heart rate (OR 1.07, 95%CI 1.04–1.10, *p* < 0.001) and waist-to-height ratio (OR 1.06, 95%CI 1.0–1.13, *p* = 0.04). The variables significantly related with cr-PWV values were diastolic BP z-score (*p* = 0.001), heart rate (*p* < 0.01), and HOMA index (*p* < 0.02). No significant association was found between the cr-PWV and BMI z-score or waist-to-height ratio. Conclusions: Systolic and diastolic BP values and central obesity are associated with aortic stiffness in a population of children and adolescents at increased cardiovascular risk. In contrast, diastolic BP, heart rate, and levels of insulin resistance appear to be related to distensibility of the upper limb vascular district.

## 1. Introduction

Measurement of pulse wave velocity (PWV) represents a simple way to measure the stiffness of a specific arterial segment [1]. The pulse wave is transmitted through the arterial vessels, and its speed is inversely related to the viscoelastic properties of the wall itself; the higher the velocity, the less elastic the wall [2]. Carotid-femoral PWV (cf-PWV) investigates the viscoelastic properties of the aorta and is considered the non-invasive gold standard for estimating the degree of aortosclerosis in daily clinical practice [3,4]. In adults, high cf-PWV values represent an independent risk factor for cardiovascular events, as well as an important prognostic factor for cardiovascular mortality [3,5]. PWV can be modified both by structural and functional elements of the arterial wall [2].

Regarding the structural elements, the viscoelastic properties of the arterial wall in large arteries are guaranteed by the ratio between the elastin fibers and the collagen fibers in the tunica media [6,7,8]. This relationship can be altered by an increase in collagen fibers (as observed in arterial hypertension), as well as by a reduction in elastic fibers (as observed with aging) [4,9,10]. The aging process causes histological alterations in the arterial wall. Reduced elastin synthesis and increased elastase activity cause thinning and breakage of elastin fibers, and the result is a decrease in the elastin and collagen ratio. Starting from the first decades of life, there is a slow but progressive increase in aortic PWV values, with a rapid and exponential increase in adults and in the elderly population [11]. If the maintenance of the structural characteristics of the arterial wall represents an important element to guarantee the viscoelastic properties of the aorta and of the large elastic arteries, on the other hand the elastin−collagen ratio in the wall has a negligible impact on the mechanical properties of the muscular arteries.

Muscular arteries are mainly affected by functional factors, mostly related to the activity of the sympathetic nervous system [2,9]. Enhanced sympathetic activity results in an increase in heart rate, ventricular contractility, and peripheral vascular resistance, leading to a rise in mean arterial pressure. Concerning arterial vessels, the sympathetic system modulates the activity and the tone of the smooth muscle cells of the arterial wall. On the other hand, the impact of the sympathetic nervous system on the distensibility properties of the aorta is weak and it has been shown that the mechanical properties of the human aorta remain largely unaffected during sympathetic stimulation. The mechanical properties of predominantly muscular peripheral arteries can be assessed in the peripheral arterial districts of the lower limbs and upper limbs, by measuring the femoral-tibial PWV and carotid-radial PWV (cr-PWV), respectively. The latter provides an estimate of the viscoelastic properties of the axillo−brachial−radial arterial district. Several studies performed on the adult population have shown that elevated femoro-tibial and cr-PWV values have no prognostic or clinical significance [12,13]. Furthermore, while cf-PWV increases significantly with aging, cr-PWV does not change significantly with age [2]. Overall, PWV assessed at the upper limb likely reflects a functional condition of the arterial tree, which is closely related to the activation of the sympathetic system.

The relationship between PWV in the aorta and in upper limb muscular arteries has not yet been investigated in childhood and adolescence, and it is unclear what factors affect cf-PWV and cr-PWV at this age in the presence of cardiovascular risk factors. Thus, the aim of our study was to evaluate the main factors associated with cf-PWV and cr-PWV values in a pediatric population at increased cardiovascular risk.

## 2. Materials and Methods

### 2.1. Participants

We studied a cohort of children and adolescents, consecutively referred from May 2008 to September 2022 to the Unit for Cardiovascular Risk Assessment in Children of Istituto Auxologico Italiano, IRCCS (Milan, Italy) by their primary care pediatricians, for the clinical finding of excess weight or elevated blood pressure (BP) values.

Children and adolescents with diabetes mellitus, secondary hypertension, hypertension under drug treatment, congenital cardiovascular disease, and kidney disease were excluded from the study. The presence of chronic disease involving habitual therapy was considered an exclusion criterion from the study.

The study protocol was approved by the local institutional ethics committee and conformed to the ethical guidelines of the 1975 Declaration of Helsinki. Informed consent was obtained from parents or legal representatives before the enrolment in the study.

### 2.2. Clinical Parameters

Height, weight, and waist circumference were measured. Waist circumference was measured by means of a flexible tape in a standing position. Body mass index (BMI) was calculated as weight/height^2^ (Kg/m^2^). The waist-to-height ratio (WtHr) was obtained dividing waist circumference by height, and expressed as percentage [14,15]. BMI z-scores were derived from the Centre for Disease and Control prevention charts [16]. All study participants were classified as normal weight, overweight, or obese according to the International Obesity Task Force classification [17]. The pubertal stage was assessed and children were divided into two categories, pre-pubertal and pubertal, according to Tanner [18,19], considering pre-pubertal boys with gonadal stage 1 and girls with breast stage 1.

### 2.3. Blood Pressure Measurement

BP measurements were performed after at least 5 min of rest, in a sitting position, using an oscillometric device validated in children (Omron 705IT; Omron Co., Kyoto, Japan) with an appropriate cuff for the upper-arm size. The BP measurement was performed 3 times (at intervals of 3 min) and the average of the last two measurements was considered. Systolic BP and diastolic BP percentiles and z-scores were calculated according to the nomograms of the National High Blood Pressure Education Program Working Group on High Blood Pressure in Children and Adolescents [20,21]. The children were classified as normotensive if both systolic and diastolic BP percentiles were <90th; high-normal if systolic BP and/or diastolic BP percentiles were ≥90th but <95th; and hypertensive if systolic BP and/or diastolic BP percentiles were ≥95th.

### 2.4. Biochemical Dosages

Fasting blood samples were taken in all study participants to measure serum glucose, insulin, uric acid, and creatinine. Commercial kits were employed for all analyses: enzymatic method with hexokinase Glucose HK Gen.3 Cobas Roche (F. Hoffmann-La Roche AG, Basel, Switzerland), for glucose assay; ElectroChemiLuminescence Elecsys Insulin Cobas Roche immunoassay was used for the insulin assay; colorimetric enzymatic test Uric Acid 2 Cobas Roche for the serum uric acid assay; and colorimetric kinetic test based on the Jaffé method Creatinine Jaffé Gen.2 Cobas Roche for creatinine assay. The homeostatic model assessment (HOMA) index was obtained by dividing the product of the serum insulin (mU/L) and serum glucose (mmol/L) by 22.5 [22]. The glomerular filtration rate was estimated (eGFR) using the Schwartz formula [23].

### 2.5. Arterial Stiffness Assessment

Measurements of arterial distensibility were obtained at a stable room temperature after 10 min of rest, by a validated ETT PulsePen tonometer [24] (DiaTecne srl, San Donato Milanese, Italy), as described in detail previously [25,26,27]. Briefly, PulsePen consists of a pocket size, high-fidelity applanation tonometer, and an integrated ECG unit. Aortic PWV was measured by recording carotid and femoral waveforms in rapid succession. cf-PWV was defined as 80% of the distance between the measuring sites divided by the time delay between the distal (femoral) pulse wave from the proximal (carotid) pulse wave, using the R wave of the ECG trace as the reference [11]. The R−R interval on the ECG recording was used to define the heart rate. The use of the PulsePen device in children had been validated in a previous study, which provided reference values according to gender and age for cf-PWV in children and adolescents [28].

### 2.6. Statistical Analysis

The characteristics of the cohort, overall and stratified according to sex, were described by median and interquartile range (Q1–Q3) if the variables were continuous and by frequencies and percentages if they were categorical. Univariate analyses to compare the characteristics of the two groups of children were conducted using the Mann−Whitney test in case of continuous variables, and through the Chi-Square test in case of categorical variables.

The univariate associations between cf-PWV (or cr-PWV) and systolic BP, diastolic BP and BMI z-score values, WtHr, uric acid, and HOMA index are represented in scatterplots, where 95% confidence interval on the Pearson correlation test and the *p*-value are displayed. 

Multiple linear regression models were used to assess the impact of sex, pubertal status, systolic BP (or diastolic BP) z-score, BMI z-score (or WtHr), heart rate (detected at the time of measurement of cf-PWV), uric acid, HOMA index, and eGFR on cf-PWV. Multiple linear regression models were used to assess the impact of sex, pubertal status, systolic BP (or diastolic BP) z-score, BMI z-score (or WtHr), heart rate (detected at the time of measurement of cf-PWV), uric acid, HOMA index, and eGFR on cr-PWV. Multiple logistic regression models were used to assess the impact of sex, pubertal status, systolic BP (or diastolic BP) z-score, BMI z-score (or WtHr), heart rate, uric acid, HOMA index, and eGFR on having cf-PWV values equal to or greater than the 95th percentile according to gender and age [28]. As there were no reference nomograms for cr-PWV, only the multiple linear regression model was performed for this variable. Statistical analyses were performed with R (R Fundation for Statistical Computing, Vienna, Austria) 4.1.2 version (http://www.R-project.org) accessed on 1 November 2023. All *p*-values were 2-sided, with *p*-values < 0.05 considered to be statistically significant.

## 3. Results

### 3.1. Population

The study involved 443 children and adolescents referred to our clinic. Table 1 shows the characteristics of the population enrolled in the study. The median age was 11.5 years; 43.3% of children were female and 54% were prepubescent. Here, 25.5% (*n* = 113) had BP values greater than or equal to the 90th percentile. Furthermore, 80.8% (*n* = 358) were of excess weight, and 67.5% (*n* = 295) had WtHr >50%. The median cf-PWV value was 4.8 m/s, and 11.4% (*n* = 50) of the children had cf-PWV values equal to or greater than the 95th percentile [28]. The cr-PWV values were significantly higher (median value 5.8 m/s) than the cf-PWV values (*p* < 0.001), without differences between males and females.

### 3.2. Factors Affecting Arterial Stiffness

**Figure 1:**
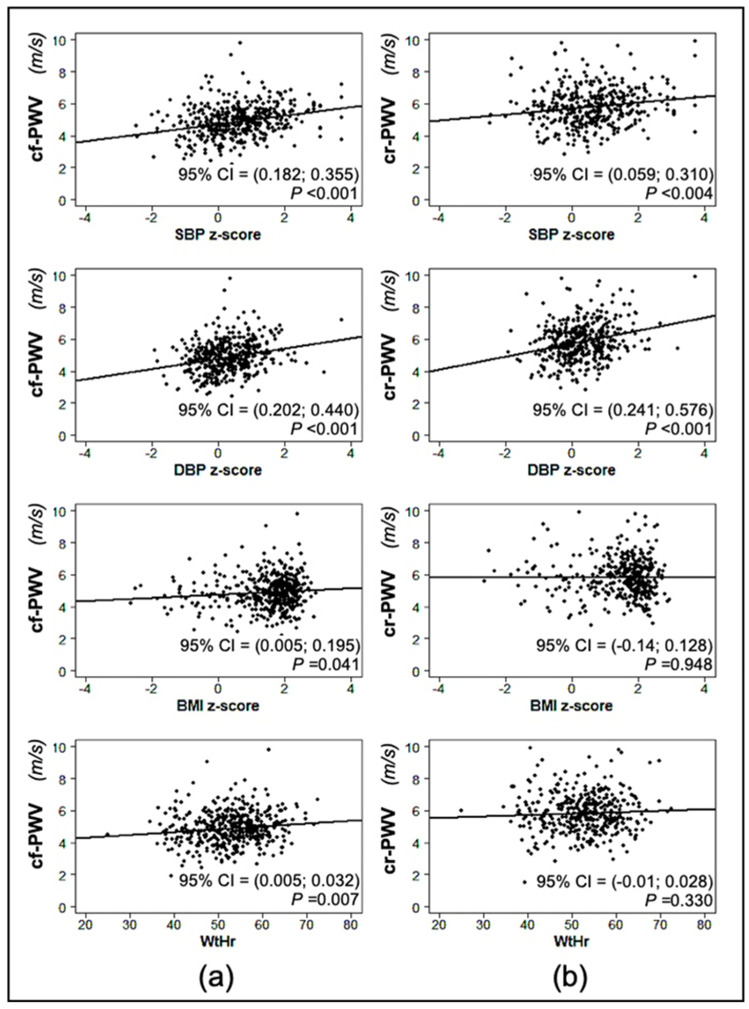
Linear regression between carotid-femoral pulse wave velocity (cf-PWV) (**a**) and carotid-radial pulse wave velocity (cr-PWV) (**b**) and systolic (SBP), diastolic blood pressure (DBP) z-score, body mass index (BMI) z-score, and waist-to-height ratio (WtHr).

**Figure 2:**
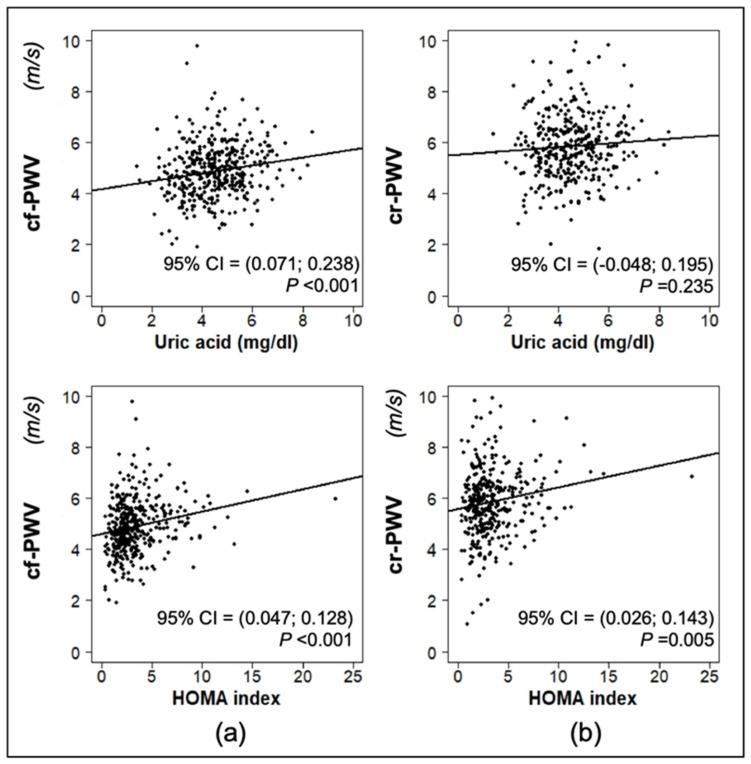
Linear regression between carotid-femoral pulse wave velocity (cf-PWV) (**a**) and carotid-radial pulse wave velocity (cr-PWV) (**b**) and serum uric acid and homeostatic model assessment (HOMA) index.

Figure 1 shows the linear regression between cf-PWV/cr-PWV values and systolic BP, diastolic BP and BMI z-scores, and WtHr. The systolic BP z-score was significantly correlated with both cf-PWV (*p* < 0.01) and cr-PWV values (*p* < 0.01). The same was true for the correlation of diastolic BP z-scores with both cf-PWV (*p* < 0.001) and cr-PWV (*p* < 0.001) values. BMI z-score and WtHr were associated with cf-PWV values (*p* = 0.041 and *p* = 0.007, respectively), but not with cr-PWV values. Both serum uric acid and HOMA index values were correlated with cf-PWV (*p* < 0.001), while only HOMA index but not serum uric acid was associated with cr-PWV (*p* = 0.005) (Figure 2).

Multiple linear regression analysis (Table 2) showed that the variables significantly associated with cf-PWV values were the presence of pubertal development (*p* < 0.03), systolic BP and diastolic BP z-scores (*p* = 0.002), and heart rate (*p* < 0.001). A correlation between HOMA index and cf-PWV was evident when the model was adjusted for diastolic BP z-score (*p* = 0.039). The estimated glomerular filtration rate (eGFR) was inversely related with cf-PWV (*p* = 0.020). No significant association was evident between BMI z-score and cf-PWV. In contrast, when WtHr was entered into the model instead of BMI z-score, a significant correlation was shown between WtHr and cf-PWV (*p* < 0.05). All of the results of the previous model were confirmed, except for the HOMA index, which was no longer associated with cf-PWV. If the HOMA index was removed from the regressors, the association between WtHr and cf-PWV became stronger (*p* < 0.01), while the other results remained unchanged.

The multiple logistic regression model exploring factors significantly associated with the presence of cf-PWV values equal to or greater than the 95th percentile for sex and age, adjusted for systolic BP z-score (Table 3), showed a direct association with heart rate (OR 1.07 95%CI 1.04–1.10, *p* < 001) and an inverse association with eGFR (OR 0.98 95% CI 0.97–0.99, *p* = 0.025) (Table 4). The results were similar when the diastolic BP z-score was included in the model. When the BMI z-score was substituted for WtHr in the model adjusted for systolic BP z-score, the OR was 1.07 (95%CI 1.04–1.10, *p* < 0.001) for heart rate and 0.98 (95%CI 0.97–0.99, *p* = 0.018) for eGFR. Interestingly, in the latter model, WtHr was also significantly associated with the presence of cf-PWV values equal to or greater than the 95th percentile (OR 1.06 95%CI 1.0–1.13, *p* = 0.040). Similar results were obtained for the model adjusted for diastolic BP z-score. The results did not change when the HOMA index was removed from the model.

Variables significantly related to cr-PWV were heart rate (*p* < 0.01) and HOMA index (*p* < 0.02), diastolic BP z-score (*p* = 0.001), but not systolic BP z-score. There was an inverse correlation between cr-PWV and eGFR (*p* = 0.035). No significant association was evident between BMI z-score and cr-PWV. When WtHr was included in the model instead of BMI z-score, the results were essentially unchanged and WtHr was not associated with cr-PWV (Table 4). The results did not change when the HOMA index was removed from the model.

## 4. Discussion

To our knowledge, this is the first study comparing parameters estimating the viscoelastic properties of the aorta and upper limb muscular arteries in a pediatric cohort with cardiovascular risk factors. As a main and innovative contribution, the present study highlights how, in this population, the factors significantly associated with upper limb arterial distensibility (estimated by cr-PWV) are somewhat different from those associated with aortic distensibility (estimated by cf-PWV). If the viscoelastic properties of muscular arteries in the upper limbs appear to be mediated by tonic levels of sympathetic activity, aortic distensibility appears, instead, to be more affected by blood pressure, heart rate, and WtHr. Interesting, a significant and inverse correlation with eGFR was found with both cf-PWV and cr-PWV values.

### 4.1. Aortic Pulse Wave Velocity

Aortic PWV depends on structural elements and transient functional changes in the arterial wall. The structural factors are stable and closely related to the relationship between elastin and collagen fibers in the tunica media of the arterial wall. The tunica media of the aorta has a typical lamellar arrangement, characterized by an orderly arrangement and interrelationship between elastic fibers and collagen fibers. Elastic fibers are characterized by an accentuated viscoelastic property and collagen fibers are mainly responsible for a structural containment function. 

The characteristically different adult patterns of elastin and collagen composition of thoracic and abdominal aortic segments are already present to some degree at birth [6]. The number of lamellar units present at birth remains almost constant in the first decade of life, then increases progressively in adulthood, doubling in the thoracic aorta (from 25–30 units to approximately 56 units in the adult), while it increases less (from 15–20 units to approximately 28 units in the adult) in the abdominal aorta [29]. BP level may be an important mechanical factor influencing the relative degree of lamellar growth during the first years of life and in childhood [29,30]. Collagen is continuously degraded and deposited in a process of homeostatic regulation [31]. An increase in BP, directly or indirectly, provides the stimulus for the elaboration of fibrous collagen proteins in the arterial wall [29], in order to counterbalance the resulting transmural pressure increase [2]. The higher synthesis of collagen fibers induced by high BP values, therefore, causes an imbalance in the elastin−collagen ratio of the arterial wall, determining a condition of aortic stiffening. This action of BP on the viscoelastic properties of the aorta explains how, in our population of children and adolescents with cardiovascular risk factors, the indexed values (z-scores) of systolic and diastolic BP and heart rate were independently associated with aortic stiffness, confirming what is already widely known in the youth [32,33] and in adult population [3,11].

A condition of aortic stiffness has been described even in metabolic diseases such as diabetes [34], fatty liver disease [35,36,37], kidney failure [12,38,39], and alterations in calcium metabolism [40,41]. Some metabolic disorders can be accompanied by an increase in oxidative stress, arterial medial calcifications, and by inflammation of the arterial wall [42]. Inflammation causes both arterial stiffening and endothelial dysfunction [43]. There is no agreement in the literature regarding the relationship between BMI and vascular stiffness in children and adolescents. Some data suggest that there is no influence of excess weight on cf-PWV [33,44], while others go in the opposite direction [45,46]. Some authors have also suggested that in obese adolescents, there is an inverse correlation between cf-PWV and BMI values. On the other hand, several studies show a close relationship between insulin resistance and arterial stiffness in children and adolescents [44,47,48,49], and this suggests that excess weight and visceral fat (related to insulin resistance) may be associated with different effects on arterial viscoelasticity, although not all authors agree on this point [50]. Our study did not show an association between cf-PWV and BMI. However, we found a significant relationship between cf-PWV and WtHr. This result is interesting, because it suggests that, for the same weight class, a greater quantity of visceral fat could lead to a more severe clinical picture, presumably related to the production of cytokines, which would induce endothelial dysfunction through an increase in oxidative stress and trigger an inflammatory process that would lead to early vascular damage [51,52]. As there is a strong relationship between central obesity and insulin resistance already in childhood [53] and the cytokines produced by visceral fat can influence BP values in children [54], it is difficult to distinguish the role of insulin resistance and/or visceral obesity when determining the viscoelastic properties of the aorta.

### 4.2. Upper Limb Pulse Wave Velocity

Along the arterial tree there is a functional diversification that corresponds to a progressive change in the composition of the arterial wall. The aorta and large elastic arteries have the characteristic lamellar structure with layers of elastin interdigitated by layers of collagen and vascular smooth muscle. These arteries contribute to the buffering function and ensure the Windkessel effect. Progressing towards the periphery of the vascular system, the arteries lose their lamellar elastic structure and evolve into muscular-type arteries with a decrease in elastin and a predominance of smooth muscle cells.

This distinction between elastic and muscular vessels is particularly important from a clinical point of view, as, if high cf-PWV values are correlated with a high cardiovascular risk, no relationship between cr-PWV and the incidence of cardiovascular disease has been demonstrated [12,13].

In healthy young subjects, the autonomic nervous system does not have a pressure-independent role in the regulation of the large elastic central arteries [55], which are little or not at all innervated by the sympathetic system [56]. On the contrary, the distal segments of the arterial tree (“muscular” arteries) are more muscular [57] and densely innervated [58,59], thus being particularly sensitive to the activity of the sympathetic system [60]. Thus, the stiffness of muscular arteries appears to be mediated by tonic levels of sympathetic activity [61]. The results of our study are in agreement with these pathophysiological premises, as upper limb PWV was independently associated with z-score of diastolic BP and heart rate, resulting from a condition of sympathetic activation.

In agreement with other studies in humans [62,63], the HOMA index (indicative of insulin resistance) also had a significant association with cr-PWV. Given the condition of sympathetic activation associated with insulin resistance, this finding also appears likely to be induced by sympathetic activity. The interpretation of data on the relationship between insulin resistance and PWV in the two vascular districts is complex. From our results, it would appear that insulin resistance has a greater role in determining carotid-radial stiffness than carotid-femoral stiffness, which, conversely, would be more influenced by central obesity. However, these findings should be interpreted with due caution and would need to be confirmed by additional studies.

### 4.3. Arterial Stiffness and Glomerular Filtration Rate

Arterial stiffness is increased in children with chronic kidney disease [64]. All children and adolescents in our study population had normal renal function. However, there was a strong inverse association between eGFR values and both cf-PWV and cr-PWV. This finding may suggest that, despite the absence of renal disease, a higher filtration rate leads to better vascular compliance. We can only speculate on the possible mechanisms behind these findings. One possibility could be that children with a higher eGFR have a smaller intravascular volume and that this may contribute to an increased vascular stiffness. Further studies are needed to test this hypothesis.

### 4.4. Study Limitations and Strenghts

While our results are supported by the consistent number of children and adolescents at increased cardiovascular risk that we were able to include in our paper, we have to acknowledge a limitation of our study, related to the fact that we were able to collect only cross-sectional data. Indeed, our hypotheses on the mechanisms behind our findings would need longitudinal data to be tested and confirmed. However, we believe that our results are, nevertheless, important, because they pave the way for such future longitudinal evaluations.

## 5. Conclusions

PWVs of the aorta and upper limb have different regulatory mechanisms and clinical significance. If the viscoelastic properties of the aorta are linked to blood pressure, heart rate and visceral fat, on the other hand the distensibility of the muscular arteries of the upper limbs seems to be mainly influenced by the sympathetic system in our population of children at increased cardiovascular risk. 

Further longitudinal studies are needed to clarify the prognostic significance of elevated cf-PWV and cr-PWV values in childhood and adolescence, as well as their possible role in the pathogenesis of arterial hypertension.

## Figures and Tables

**Table 1:** Anthropometric and clinical characteristics according to sex.

Parameter	Overall	Females	Males	*p*-Value
Participants, *n* (%)	443	192 (43.3)	251 (56.7)	
Age, years	11.5 (9.3–13.2)	10.9 (8.7–13.0)	11.7 (9.9–13.5)	0.005
Birth weight, g	3280 (2900–3640)	3200 (2800–3530)	3300 (3000–3800)	0.004
Puberty, *n* (%)	201 (46.0)	91 (47.6)	110 (44.7)	0.608
Heart rate, beats/min	76 (69–85)	80 (72–87)	73 (66–82)	<0.001
Systolic BP, mmHg	111 (103–121)	109 (101–119)	112 (104–123)	0.010
Systolic BP z-score	0.58 (−0.12–1.21)	0.66 (−0.16–1.18)	0.53 (−0.09–1.27)	0.999
Diastolic BP, mmHg	65 (59–71)	65 (58–71)	64 (60–71)	0.685
Diastolic BP z-score	0.21 (−0.25–0.81)	0.31 (−0.23–0.85)	0.17 (−0.25–0.76)	0.372
BP category:				0.752
- Normotension, *n* (%)	330 (74.5)	144 (75.0)	186 (74.1)	
- High-normal, *n* (%)	42 (9.5)	16 (8.3)	26 (10.4)	
- Hypertension, *n* (%)	71 (16.0)	32 (16.7)	39 (15.5)	
Weight class:				0.771
- Normal weight, *n* (%)	85 (19.2)	39 (20.3)	46 (18.3)	
- Overweight, *n* (%)	141 (31.8)	58 (30.2)	83 (33.1)	
- Obese, *n* (%)	217 (49.0)	95 (49.5)	122 (48.6)	
BMI, Kg/m^2^	24.6 (21.8–27.7)	24.2 (20.9–26.9)	25.1 (22.5–28.1)	0.010
BMI z-score	1.78 (1.20–2.11)	1.76 (1.10–2.04)	1.81 (1.32–2.17)	0.087
Waist-to-height ratio, %	53.3 (48.3–57.8)	52.4 (47.3–57.3)	54.0 (49.4–57.9)	0.021
Waist-to-height ratio >50%, %	295 (67.5)	119 (63.3)	176 (70.7)	0.126
Serum uric acid, mg/dl	4.5 (3.7–5.3)	4.4 (3.7–4.9)	4.7 (3.7–5.6)	0.003
Glucose, mg/dl	86 (81–89)	85 (80–89)	86 (82–89)	0.072
Insulin, mM/L	13.0 (9.0–18.6)	12.9 (9.2–18.4)	13.0 (9.0–18.7)	0.866
HOMA index, mmol/L × mU/L	2.6 (1.9–4.0)	2.6 (1.9–3.9)	2.7 (1.9–4.1)	0.944
Creatinine, mg/dl	0.54 (0.48–0.63)	0.52 (0.45–0.60)	0.57 (0.50–0.66)	0.003
eGFR, ml/min	149 (132–164)	151 (134–171)	147 (129–161)	0.046
cf-PWV, m/s	4.8 (4.3–5.5)	4.8 (4.3–5.5)	4.8 (4.3–5.5)	0.752
cf-PWV ≥ 95th percentile, *n* (%)	50 (11.4)	22 (11.6)	28 (11.3)	0.999
cr-PWV, m/s	5.8 (5.0–6.5)	5.8 (5.3–6.5)	5.6 (4.9–6.4)	0.069

Data are shown as median (interquartile range) or number (%). BMI, body mass index; BP, blood pressure; cf-PWV, carotid-femoral pulse wave velocity; cr-PWV, carotid-radial pulse wave velocity; eGFR, estimated glomerular filtration rate; HOMA, homeostatic model assessment.

**Table 2:** Results of multiple linear regression analysis with carotid-femoral pulse wave velocity (m/s) as dependent variables in the entire sample.

**Dependent Variable: Carotid-Femoral Pulse Wave Velocity**						
**I Analysis**						
**Variable**	**Model A**			**Model B**		
	**β**	**(95% CI)**	***p***	**β**	**(95% CI)**	***p***
Intercept	3.772	(2.767; 4.778)	<0.001	3.795	(2.788; 4.801)	<0.001
Sex (males)	0.066	(−0.135; 0.268)	0.517	0.076	(−0.125; 0.278)	0.456
Puberty	0.263	(0.038; 0.487)	0.022	0.312	(0.091; 0.533)	0.006
Systolic BP z-score	0.161	(0.059; 0.262)	0.002	-	-	-
Diastolic BP z-score	-	-	-	0.219	(0.084; 0.355)	0.002
BMI z-score	0.081	(−0.033; 0.195)	0.163	0.081	(−0.033; 0.195)	0.162
Heart rate, beats/min	0.018	(0.010; 0.026)	<0.001	0.017	(0.009; 0.026)	<0.001
Serum uric acid, mg/dL	−0.005	(−0.106; 0.096)	0.925	−0.003	(−0.105; 0.098)	0.947
HOMA index	0.044	(−0.001; 0.090)	0.057	0.048	(0.002; 0.093)	0.039
eGFR, mL/min	−0.005	(−0.009; −0.001)	0.020	−0.005	(−0.009; −0.001)	0.021
**II Analysis**						
**Variable**	**Model A**			**Model B**		
	**β**	**(95% CI)**	***p***	**β**	**(95% CI)**	***p***
Intercept	3.018	(1.839; 4.198)	<0.001	3.085	(1.898; 4.273)	<0.001
Sex (males)	0.052	(−0.152; 0.256)	0.616	0.061	(−0.143; 0.265)	0.557
Puberty	0.307	(0.074; 0.541)	0.010	0.350	(0.120; 0.581)	0.003
Systolic BP z-score	0.161	(0.059; 0.262)	0.002	-	-	-
Diastolic BP z-score	-	-	-	0.213	(0.075; 0.351)	0.003
WtHr	0.019	(0.003; 0.036)	0.022	0.018	(0.002; 0.035)	0.032
Heart rate, beats/min	0.018	(0.009; 0.026)	<0.001	0.017	(0.008; 0.025)	<0.001
Serum uric acid, mg/dL	−0.015	(−0.117; 0.086)	0.771	−0.011	(−0.112; 0.091)	0.836
HOMA index	0.035	(−0.013; 0.082)	0.150	0.039	(−0.008; 0.086)	0.102
eGFR, mL/min	−0.005	(−0.010; −0.001)	0.015	−0.005	(−0.010; −0.001)	0.016

In Model A and Model B, systolic blood pressure or diastolic blood pressure were considered, respectively. In Analysis I and Analysis II, body mass index (z-score) or waist-to-height ratio were considered, respectively. Coefficient β provides a measure of the relative strength of the association independent of the units of measurement. BMI, body mass index; BP, blood pressure; CI, confidence interval; eGFR, estimated glomerular filtration rate; HOMA, homeostatic model assessment; WtHr, waist-to-height ratio.

**Table 3:** Results of multiple regression model with carotid-femoral pulse wave velocity values equal to or greater than the 95th percentile as dependent variables.

**Dependent Variable: Carotid-Femoral Pulse Wave Velocity Equal to or Greater than the 95th Percentile**						
**I Analysis**						
**Variable**	**Model A**			**Model B**		
	**β**	**(95% CI)**	***p***	**β**	**(95% CI)**	***p***
Sex (males)	1.448	(0.746; 2.866)	0.279	1.489	(0.765; 2.962)	0.247
Puberty	0.732	(0.342; 1.532)	0.413	0.690	(0.326; 1.425)	0.322
Systolic BP z-score	0.898	(0.632; 1.268)	0.545	-	-	-
Diastolic BP z-score	-	-	-	1.283	(0.825; 1.981)	0.262
BMI z-score	1.289	(0.884; 1.959)	0.206	1.244	(0.853; 1.891)	0.277
Heart rate, beats/min	1.068	(1.039; 1.099)	<0.001	1.065	(1.036; 1.096)	<0.001
Serum uric acid, mg/dL	0.774	(0.541; 1.095)	0.153	0.756	(0.530; 1.067)	0.116
HOMA index	0.964	(0.793; 1.137)	0.689	0.937	(0.771; 1.107)	0.482
eGFR, mL/min	0.982	(0.967; 0.997)	0.018	0.983	(0.967; 0.997)	0.025
**II Analysis**						
**Variable**	**Model A**			**Model B**		
	**β**	**(95% CI)**	***p***	**β**	**(95% CI)**	***p***
Sex (males)	1.489	(0.758; 2.996)	0.254	1.500	(0.762; 3.025)	0.247
Puberty	0.936	(0.420; 2.059)	0.870	0.872	(0.395; 1.893)	0.730
Systolic BP z-score	0.867	(0.604; 1.235)	0.431	-	-	-
Diastolic BP z-score	-	-	-	1.225	(0.773; 1.916)	0.379
WtHr	1.064	(1.004; 1.130)	0.040	1.060	(1.001; 1.126)	0.050
Heart rate, beats/min	1.069	(1.039; 1.100)	<0.001	1.066	(1.036; 1.097)	<0.001
Serum uric acid, mg/dL	0.711	(0.488; 1.020)	0.070	0.697	(0.480; 0.995)	0.051
HOMA index	0.941	(0.764; 1.123)	0.538	0.910	(0.739; 1.089)	0.340
eGFR, mL/min	0.982	(0.966; 0.996)	0.018	0.982	(0.967; 0.997)	0.022

In Model A and Model B, systolic blood pressure or diastolic blood pressure were considered, respectively. In Analysis I and Analysis II, body mass index (z-score) or waist-to-height ratio were considered, respectively. BMI, body mass index; BP, blood pressure; CI, confidence interval; eGFR, estimated glomerular filtration rate; HOMA, homeostatic model assessment; OR, odds ratio; WtHr, waist-to-height ratio.

**Table 4:** Results of multiple linear regression analysis with carotid-radial pulse wave velocity (m/s) as dependent variables in the entire sample.

**Dependent Variable: Carotid-Radial Pulse Wave Velocity**						
**I Analysis**						
**Variable**	**Model A**			**Model B**		
	**β**	**(95% CI)**	***p***	**β**	**(95% CI)**	***p***
Intercept	5.035	(3.463; 6.607)	<0.001	5.240	(3.688; 6.791)	<0.001
Sex (males)	−0.164	(−0.467; 0.139)	0.288	−0.138	(−0.438; 0.161)	0.365
Puberty	0.017	(−0.318; 0.351)	0.922	0.033	(−0.292; 0.358)	0.842
Systolic BP z-score	0.076	(−0.077; 0.230)	0.327	-	-	-
Diastolic BP z-score	-	-	-	0.332	(0.131; 0.533)	0.001
BMI z-score	−0.066	(−0.234; 0.103)	0.444	−0.094	(−0.260; 0.073)	0.269
Heart rate, beats/min	0.022	(0.009; 0.035)	0.001	0.019	(0.006; 0.032)	0.004
Serum uric acid, mg/dL	0.015	(−0.137; 0.167)	0.845	−0.001	(−0.152; 0.149)	0.985
HOMA index	0.087	(0.019; 0.155)	0.012	0.080	(0.013; 0.146)	0.019
eGFR, mL/min	−0.007	(−0.014; −0.001)	0.022	−0.007	(−0.013; 0.000)	0.035
**II Analysis**						
**Variable**	**Model A**			**Model B**		
	**β**	**(95% CI)**	***p***	**β**	**(95% CI)**	***p***
Intercept	4.980	(3.157; 6.802)	<0.001	5.385	(3.579; 7.190)	<0.001
Sex (males)	−0.186	(−0.494; 0.121)	0.235	−0.166	(−0.470; 0.138)	0.283
Puberty	0.042	(−0.308; 0.393)	0.813	0.045	(−0.296; 0.386)	0.796
Systolic BP z-score	0.071	(−0.083; 0.225)	0.364	-	-	-
Diastolic BP z-score	-	-	-	0.329	(0.125; 0.534)	0.002
WtHr	0.004	(−0.020; 0.029)	0.728	0.000	(−0.024; 0.025)	0.992
Heart rate, beats/min	0.021	(0.008; 0.034)	0.002	0.018	(0.005; 0.031)	0.008
Serum uric acid, mg/dL	0.001	(−0.152; 0.154)	0.993	−0.015	(−0.166; 0.136)	0.844
HOMA index	0.076	(0.005; 0.147)	0.037	0.068	(−0.001; 0.138)	0.055
eGFR, mL/min	−0.008	(−0.014; −0.001)	0.016	−0.007	(−0.014; −0.001)	0.025

In Model A and Model B, systolic blood pressure or diastolic blood pressure were considered, respectively. In Analysis I and Analysis II, body mass index (z-score) or waist-to-height ratio were considered, respectively. The coefficient β provides a measure of the relative strength of the association independent of the units of measurement. BMI, body mass index; BP, blood pressure; CI, confidence interval; eGFR, estimated glomerular filtration rate; HOMA, homeostatic model assessment; WtHr, waist-to-height ratio.

## Data Availability

The data presented in this study are available upon reasonable request from the corresponding author. The data are not publicly available due to privacy concerns.
